# Age-Specific Cutoff Value for the Application of Percent Free Prostate-Specific Antigen (PSA) in Chinese Men with Serum PSA Levels of 4.0–10.0 ng/ml

**DOI:** 10.1371/journal.pone.0130308

**Published:** 2015-06-19

**Authors:** Rui Chen, Yiran Huang, Xiaobing Cai, Liping Xie, Dalin He, Liqun Zhou, Chuanliang Xu, Xu Gao, Shancheng Ren, Fubo Wang, Lulin Ma, Qiang Wei, Changjun Yin, Ye Tian, Zhongquan Sun, Qiang Fu, Qiang Ding, Junhua Zheng, Zhangqun Ye, Dingwei Ye, Danfeng Xu, Jianquan Hou, Kexin Xu, Jianlin Yuan, Xin Gao, Chunxiao Liu, Tiejun Pan, Yinghao Sun, Chinese Prostate Cancer Consortium

**Affiliations:** 1 Department of Urology, Shanghai Changhai Hospital, Second Military Medical University, No. 168 Changhai Road, Shanghai, 200433, China; 2 Renji Hospital, Shanghai Jiao Tong University, School of Medicine, No.1630 Dongfang Road, Shanghai, 200127, China; 3 Department of Health Care, PLA Headquarters of the General Staff Guard Bureau, Beijing 100017, China; 4 Department of Urology, First Affiliated Hospital, School of Medicine, Zhejiang University, Qingchun Road 79, Hangzhou 310003, Zhejiang Province, China; 5 Department of Urology, First Affiliated Hospital of Medical School, Xi'an Jiaotong University, No.277 west Yanta Road, Xi'an 710061, China; 6 Department of Urology, Peking University First Hospital, Institute of Urology, Peking University, National Urological Cancer center, 8 Xishiku Street, Xicheng District, Beijing 100034, China; 7 Department of Urology, Peking University Third Hospital, No 49,North Garden Road, Haidian Dist, Beijing, China; 8 Department of Urology, West China Hospital, Sichuan University, Guoxuexiang 37# 610041, Chengdu Sichuan, China; 9 State Key Laboratory of Reproductive Medicine, The First Affiliated Hospital of Nanjing Medical University, Nanjing, China; No.300 Guangzhou Road, Nanjing, Jiangsu Province, China; 10 Department of Urology, The First Affiliated Hospital of Nanjing Medical University, Nanjing, China; No.300 Guangzhou Road, Nanjing, Jiangsu Province, China; 11 Department of Urology, Beijing Friendship Hospital, Capital Medical University, Beijing, 100050, China; 12 Department of Urology, Huadong Hospital, Fudan University, #221 West Yan’an Road, Shanghai 20040, China; 13 Shanghai Jiao Tong University Affiliated Sixth People’s Hospital, Yishan Rd.600 Shanghai, China; 14 Huashan Hospital, Fudan University, No. 12, WuLuMuQi Middle Road, Shanghai, 200040, China; 15 Department of Urology, Tenth People's Hospital; Tongji University, Shanghai, China; 16 Department of Urology, Tongji Hospital, Tongji Medical College, Huazhong University of Science and Technology, 1095 Jiefang Ave. Wuhan, 430030, China; 17 Fudan University Shanghai Cancer Center and Department of Oncology, #270 Dong An Road, Shanghai, 200032, China; 18 Department of Urology, Shanghai Changzheng Hospital, Second Military Medical University, Shanghai, China; 19 The First Affiliated Hospital of Soochow University, 188 ShiZhi Road, Suzhou, 215006, China; 20 Peking University People's Hospital, 11 Xizhomen South Street, Beijing, 100044, China; 21 Department of Urology, Xijing Hospital, The Fourth Military Medical University, 127 Changlexi Road, Xi'an, 710032 China; 22 Department of Urology, 3rd Hospital of Sun Yat-Sen University, Tiehe Road 600#, Guangzhou, 510630, China; 23 Department of Urology, Zhujiang Hospital, Southern Medical University, No. 253 Industrial Road, Haizhu District, Guangzhou, Guangdong, 510282, China; 24 Department of Urology, Wuhan General Hospital of Guangzhou Military Command, No. 627 Wuluo Rd, Wuhan, Hubei 430070, China; National Health Research Institutes, TAIWAN

## Abstract

**Objective:**

The influence of age on the performance of percent free prostate-specific antigen (%fPSA) in diagnosing prostate cancer (PCa) in East Asians is controversial. We tested the diagnostic performance of %fPSA in a multi-center biopsy cohort in China and identified the proper age-specific cutoff values to avoid unnecessary biopsies.

**Methods:**

Consecutive patients with a prostate-specific antigen (PSA) level of 4.0–10.0 ng/ml or 10.1–20.0 ng/ml who underwent transrectal ultrasound-guided or transperineal prostate biopsy were enrolled from 22 Chinese medical centers from Jan 1, 2010 to Dec 31, 2013. The diagnostic accuracy of PSA and %fPSA was determined using the area under the receiver operating characteristic (ROC) curve (AUC). Age-specific cutoff values were calculated using ROC curve analysis.

**Results:**

The median %fPSA was much lower in younger patients compared with older patients with a PSA level of 4.0–10.0 ng/ml or 10.1–20.0 ng/ml. The AUC of %fPSA was higher than PSA only in older patients. In patients aged 50 to 59 years, %fPSA failed to improve the diagnosis compared with PSA in these two PSA ranges. Age-specific cutoff values were 24%, 27% and 32% for patients aged 60–69, 70–79 and ≥80 years, respectively, to reduce unnecessary biopsies in men with PSA levels of 4.0–10.0 ng/ml to detect 90% of all PCa.

**Conclusions:**

The effectiveness of %fPSA is correlated with age in the Chinese population. Age-specific cutoff values would help avoid unnecessary biopsies in the Chinese population.

## Introduction

Prostate cancer (PCa) is the second most common diagnosed malignancy in males globally [1]. Although its incidence in China is much lower than Western countries [2], PCa has become the fastest-growing malignancy in recent years [3, 4] for multiple reasons. Other biomarkers are emerging, but PSA and its derivatives remain the most widely used and clinically practical test for PCa detection. Percent free PSA (%fPSA) is recommended to reduce unnecessary biopsies for men with serum prostate-specific antigen (PSA) levels of 4.0–10.0 ng/ml in white and black populations [5, 6].

Oesterling et al. [7] established age-specific reference ranges for serum PSA levels in Caucasians to improve its sensitivity in younger patients and to increase its specificity in older patients. Age-specific cutoff values were established in African [8] and Asian populations because of differences between races [8]. Our previous studies found that age-specific reference ranges of PSA are lower in Chinese populations [9]. However, the relationship between age and %fPSA has been less studied and reported. Beggers and colleagues [10] indicated that %fPSA increases with age and proposed age-specific reference ranges of %fPSA in patients aged 40 to 79 years. In clinical trials, Catalona et al. [5] observed different levels of %fPSA in different age groups. However, these age-specific cutoff values were not recommended because the 25% cutoff yielded sensitivities of 90%, 92% and 96% in patients aged 50–59, 60–69 and 70–75 years, respectively. More than 90% of cancers will be detected and more unnecessary biopsies will be avoided. Furthermore, the 25% cutoff yielded a higher sensitivity in younger groups than would be favorable for these patients because they benefit more from the diagnosis of PCa.

The diagnostic efficacy of %fPSA is weaker in Chinese and Korean populations than Western populations [11, 12]. Some studies excluded %fPSA from multivariable nomograms for PCa prediction in the Chinese population [13]. In our previous studies we have identified that %fPSA is not more effective than PSA in Chinese men aged 40–59 years with a PSA of 4.0–10.0 ng/ml [14]. However, the results were not easy to be applied in a clinical scenario to reduce necessary biopsies. To translate the results from the AUC in ROC curve analysis to a binary and clinical applicable test, we tried to identify the age-specific cutoff values to reduces unnecessary biopsies in a multi-center Chinese cohort with a PSA level of 4.0–10.0 ng/ml or 10.0–20.0 ng/ml.

## Methods

### Patients

The Institutional Review Board of Shanghai Changhai Hospital approved this study. Written informed consent was obtained from the participants. This retrospective study consisted of a total of 5915 consecutive patients who underwent initial transrectal ultrasound-guided (TRUS-guided) biopsy or transperineal prostate biopsy from Jan 1, 2010 to Dec 31, 2013 in the 22 participating hospitals. All patients were enrolled at the outpatient Department of Urology with/without urinary symptoms. Patients were recommended to undergo biopsy as long as abnormal PSA and/or abnormal digital rectal examination (DRE) results were obtained. A few patients (1.7%) who were not willing to undergo this procedure were completely informed of intensive follow-up, and they were not included in this study. Exclusion criteria were urinary tract infections, urinary retention, recent instrumentation or catheterization of the urethra, and finasteride or hormonal treatment.

### PSA measurement and biopsy techniques

Peripheral blood samples were obtained within two weeks prior to DRE and prostate biopsy. Prostate volume (PV) was calculated using the equation D1*D2*D3*(π/6), and the three dimensions of the prostate were measured by TRUS. Three types of PSA/fPSA electro-chemiluminescence immunoassays were used in the participating hospitals (Abbott AxSYM, Beckman Coulter Access and Roche Elecsys 2010). Recalibration was performed to WHO standards (PSA-WHO 96/670) using an appropriate correction factor.

### Statistical analysis

The Mann-Whitney U test was used to analyze PSA, %fPSA, PV, and age. Simple linear regression analyses were used to evaluate the correlation between clinical parameters and %fPSA. Univariate logistic regression analyses were used to assess correlations between clinical parameters and biopsy results. Clinical parameters that were significantly associated with biopsy results were further tested using multivariate logistic regression analyses. The demographic and clinical variables used in the model included age, PSA, %fPSA and prostate volume. Parameters were included and excluded in a stepwise manner (α<0.05 for inclusion and α<0.1 for exclusion). We further combined PSA and %fPSA using logistic regression to generate a new parameter (%fPSA + PSA) and estimate the influence of age on the performance of the combination of these two parameters. Receiver operating characteristic (ROC) curves were calculated for PSA and %fPSA by plotting the sensitivity versus (1 – specificity) for predicting PCa. The areas under the ROC curves (AUC) were used to measure the diagnostic accuracy of PSA and %fPSA. The statistical significance of any difference was calculated using the z test. Sensitivity, specificity, positive predicted value (PPV) and negative predicted value (NPV) were calculated at all possible cutoff values in the application of %fPSA to diagnose PCa. All of the statistical analyses were performed using the Statistical Package for Social Science (SPSS), v.17.0 (SPSS Inc., Chicago, IL, USA) and MedCalc v.10.4.7.0 (MedCalc Software bvba, Mariakerke, Belgium). All of the p-values were two-sided, and p<0.05 was considered statistically significant.

## Results

### Patients and clinical characteristics

Detection rates of patients with PSA levels of 4.0–10.0 ng/ml or 10.0 to 20.0 ng/ml were 25.3% and 36.5%, respectively. The median age of PCa patients was 71 and 70 years in the PSA 4.0–10.0 ng/ml and 10.1–20.0 ng/ml groups, respectively, which were higher than men with negative biopsies (66 and 66 years, p<0.0001 and p<0.0001) (Table 1). Total PSA, PV and the number of biopsy cores were also significantly different between PCa patients and negative biopsies in both PSA ranges. Comparisons between the two PSA ranges showed significant differences in all of these variables except the number of biopsy cores.

**Table 1:** Clinical variables in prostate cancer and non-prostate cancer subjects in two PSA ranges.

	PSA 4–10 ng/ml			PSA 10.1–20 ng/ml			P#
	Prostate cancer	Negative Biopsy	P	Prostate cancer	Negative Biopsy	P	
No. of subjects (n)	796	2365		1003	1751		
Age							
Mean (SD)	69.9(7.7)	66.1(9.1)		70.0(7.7)	66.1(9.1)		
Median (IQR)	71(65–76)	66(60–73)	<0.0001*	71(65–76)	66(60–73)	<0.0001*	< 0.0001
PSA							
Mean (SD)	7.3(1.6)	7.1(1.7)		7.3(1.6)	7.1(2.7)		
Median (IQR)	7.4(6.0–8.7)	7.1(5.7–8.5)	0.004 *	7.4(6.0–8.6)	7.1(5.4–8.4)	0.003*	< 0.0001
Percent free PSA, (%)							
Mean (SD)	15.4(8.0)	16.6(8.1)		15.4(8.1)	16.6(8.1)		
Median (IQR)	14.0(10.0–19.0)	15.3(10.9–21.0)	<0.0001*	14.0(10.0–19.0)	15.2(10.9–21.0)	<0.0001*	< 0.0001
Prostate volume							
Mean (SD)	42.9(22.8)	51.2(27.2)		43.0(22.8)	51.2(27.3)		
Median (IQR)	37.0(27.6–52.0)	44.9(32.0–63.6)	<0.0001*	37.0(27.7–52.0)	44.9(32.3–63.7)	<0.0001*	< 0.0001
No. of biopsies Cores							
Mean (SD)	10.7(2.4)	10.4(2.3)		10.7(2.4)	10.5(2.3)		
Median (IQR)	12(9–12)	10(8–12)	0.001 *	12(9–12)	10(8–12)	0.001*	0.83

No. of subjects: number of subjects; SD: standard deviation; IQR: interquartile range; PSA: prostate-specific antigen

* Mann-Whitney U test, comparisons between prostate cancer and negative biopsy

# Mann-Whitney U test, comparisons between the two PSA ranges.

### Impact of age on %fPSA

Simple linear regression analyses indicated that age was positively correlated with %fPSA in patients with PSA levels of 4.0–10.0 ng/ml or 10.1–20.0 ng/ml (R^2^ = 0.067, p<0.001 and R^2^ = 0.039, p<0.001, respectively). The median %fPSA ranged from 12.0% in patients aged 50–59 years to 17.2% in patients aged 70–79 years and 15.7% in patients aged over 80 years in the PSA range of 4.0–10.0 ng/ml (Table 2).

**Table 2:** Diagnostic accuracy of total PSA and %fPSA in predicting prostate cancer and high-grade prostate cancer stratified by age decades.

Diagnosis Utility	Age,	No. Pts	%fPSA, Median(IQR)	AUC			P*	P#	No. Pts	%fPSA, Median(IQR)	AUC			P*	P#
	(years)			PSA	%fPSA	PSA+%fPSA					PSA	%fPSA	PSA+%fPSA		
		PSA 4.0–10.0ng/ml							PSA 10.1–20.0ng/ml						
Predict PCa	50–59	470	12.0 (8.9–16.0)	0.526 (0.457 - 0.595)	0.561 (0.492–0.630)	0.552(0.482–0.621)	0.430	0.293	283	12.0 (8.0–17.0)	0.530 (0.442 - 0.617)	0.567 (0.486 - 0.648)	0.573(0.494–0.652)	0.554	0.334
	60–69	1193	15.0 (11.0–20.0)	0.545 (0.505–0.585)	0.592 (0.552–0.631)	0.598(0.557–0.638)	0.080	0.001	961	13.2 (9.4–18.6)	0.542 (0.504 - 0.581)	0.650 (0.612 - 0.688)	0.638(0.600–0.675)	< 0.001	< 0.001
	70–79	1051	17.2 (11.9–23.0)	0.520 (0.483–0.557)	0.594 (0.558–0.631)	0.579(0.543–0.615)	0.004	<0.001	1048	14.8 (10.0–20.4)	0.531 (0.495–0.567)	0.632 (0.597–0.666)	0.628(0.593–0.662)	< 0.001	< 0.001
	80–89	190	15.7 (11.0–23.5)	0.550 (0.530–0.697)	0.613 (0.466–0.634)	0.614(0.531–0.697)	0.253	0.067	279	15.8 (10.9–22.0)	0.599 (0.533–0.666)	0.685 (0.622–0.747)	0.693(0.632–0.755)	0.064	0.006
Predict HGPCa	50–59	470	12.0 (8.9–16.0)	0.563(0.479–0.646)	0.552(0.462–0.642)	0.494(0.406–0.581)	0.868	0.220	283	12.0 (8.0–17.0)	0.515(0.415–0.616)	0.599(0.508–0.691)	0.598(0.508–0.688)	0.221	0.099
	60–69	1193	15.0 (11.0–20.0)	0.546(0.494–0.598)	0.574(0.522–0.626)	0.584(0.532–0.636)	0.432	0.122	961	13.2 (9.4–18.6)	0.555(0.512–0.598)	0.688(0.648–0.729)	0.679(0.639–0.719)	< 0.001	< 0.001
	70–79	1051	17.2 (11.9–23.0)	0.531(0.490–0.572)	0.610(0.570–0.651)	0.605 (0.565–0.646)	0.006	< 0.001	1048	14.8 (10.0–20.4)	0.544(0.505–0.584)	0.626(0.589–0.662)	0.633(0.597–0.670)	0.003	< 0.001
	80–89	190	15.7 (11.0–23.5)	0.564(0.467–0.662)	0.628(0.539–0.717)	0.643(0.554–0.733)	0.308	0.071	279	15.8 (10.9–22.0)	0.593(0.523–0.664)	0.633(0.566–0.700)	0.651(0.586–0.716)	0.431	0.129

No. Pts: number of patients.

* z test for comparing PSA vs. %fPSA.

# z test for comparing PSA vs. PSA+%fPSA

### Impact of age on the diagnostic performance of %fPSA in predicting any PCa

Univariate logistic regression analyses indicated that older age, higher PSA, lower %fPSA, and smaller PV were correlated with a positive biopsy result. Multivariate logistic regression analyses indicated that lower %fPSA, older age, higher PSA and smaller PV were independent predictors of PCa in patients with PSA 4.0–10.0 ng/ml (p<0.0001, p = 0.009, p<0.011 and p<0.0001, respectively) and PSA 10.1–20.0 ng/ml (p<0.0001, p<0.0001, p = 0.0001 and p<0.0001, respectively). Results of stratified analysis by age, prostate volume, biopsy scheme and biopsy path are summarized in Table 2 and S1 Table. The influence of age dominated, whereas the influence of other parameters only slightly changed the results of the overall analysis.

**Fig 1:**
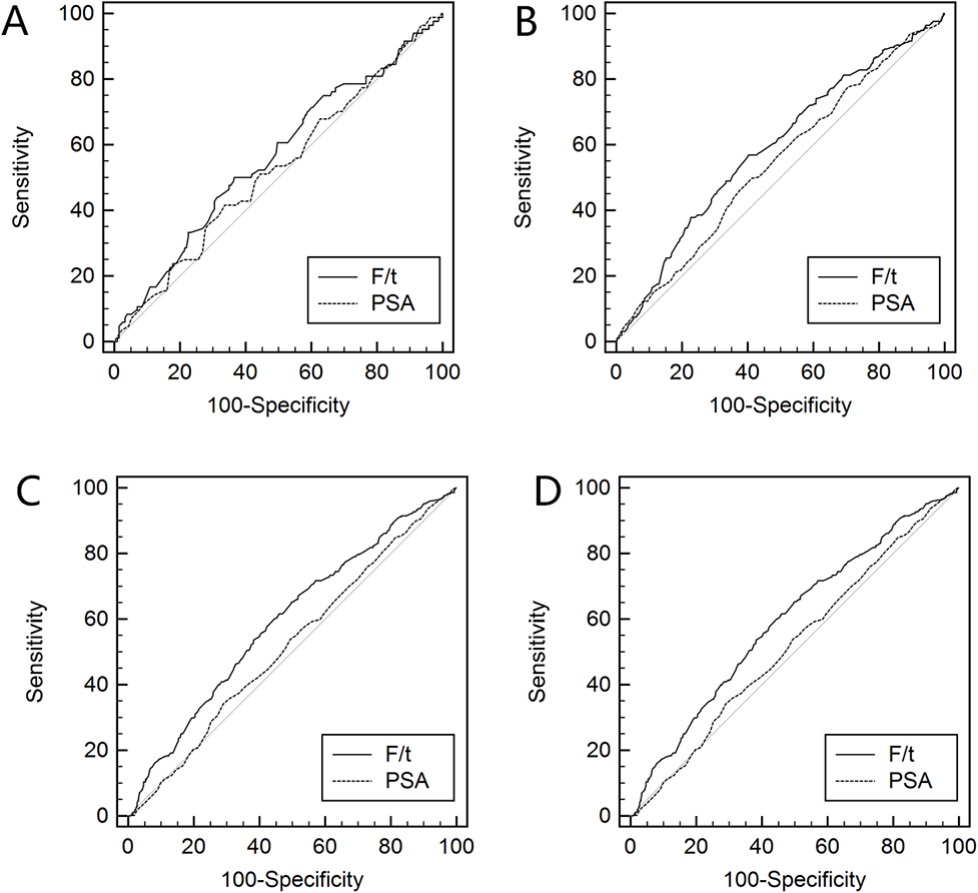
ROC curves of %fPSA and PSA for any PCa. ROC curves of %fPSA and PSA in predicting any prostate cancer for patients aged (A) 50–59 years; (B) 60–69 years; (C) 70–79 years; and (D) 80–89 years with a PSA level of 4.0–10.0 ng/ml.

**Fig 2:**
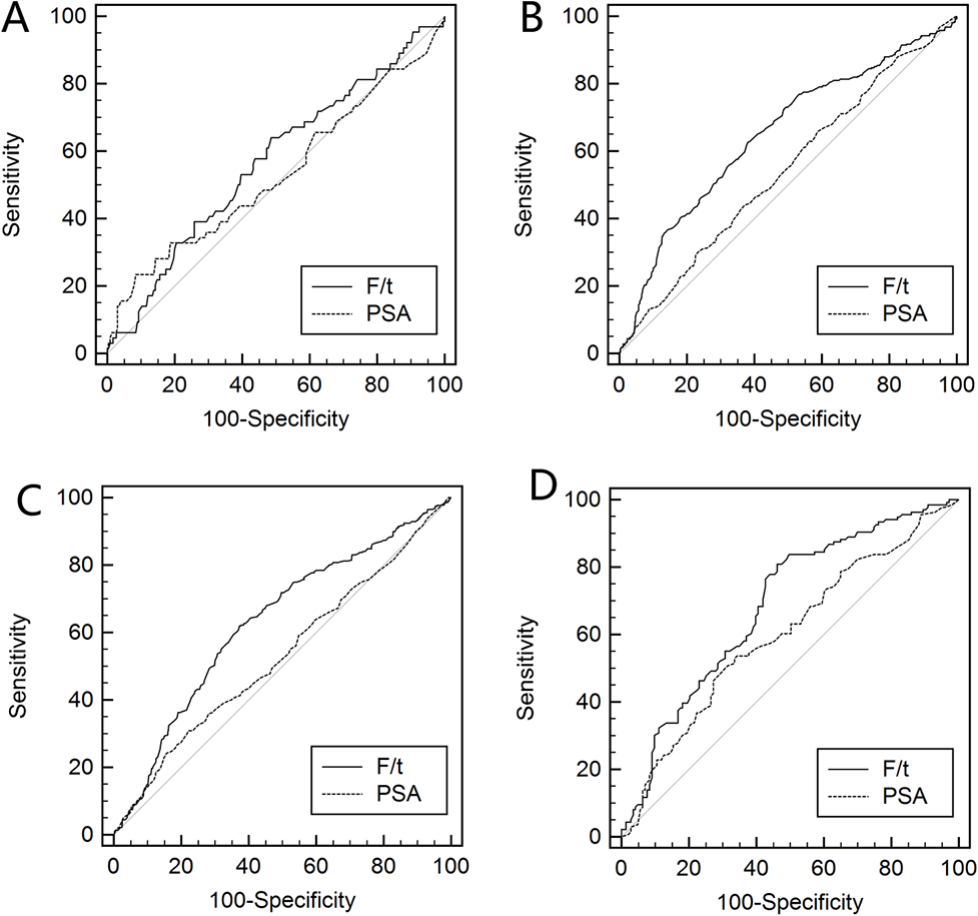
ROC curves of %fPSA and PSA for high-grade PCa. ROC curves of %fPSA and PSA in predicting high-grade prostate cancer for patients aged (A) 50–59 years; (B) 60–69 years; (C) 70–79 years; and (D) 80–89 years with a PSA level of 4.0–10.0 ng/ml.

The diagnostic performances of %fPSA and PSA in different age decades are also illustrated in the ROC curves in Fig 1 (PSA range 4.0–10.0 ng/ml) and Fig 2 (PSA range 10.1–20.0 ng/ml). %fPSA failed to improve diagnosis in patients aged 50–59 years compared with patients with PSA levels of 4.0–10.0 ng/ml or 10.1–20.0 ng/ml (p = 0.43 and 0.55, respectively) (Table 2). However, %fPSA improved AUC significantly in patients aged 60–69 years with PSA levels of 4.0–10.0 ng/ml or 10.1–20.0 ng/ml. The improvement in %fPSA failed to reach statistical significance (p = 0.08). %fPSA significantly outperformed PSA in AUC in patients aged 70–79 years old with PSA levels of 4.0–10.0 ng/ml or 10.1–20.0 ng/ml. The AUC of %fPSA was higher in patients aged 80–89 years than PSA in men with a PSA level of 10.0–20.0 ng/ml or 4.0–10.0 ng/ml, but these differences failed to reach statistical significance (p = 0.064 and 0.253).

The diagnostic accuracy of %fPSA in patients who underwent extended biopsy (10- or 12-core-based biopsy) was not higher than PSA in patients aged 50–59 years with a PSA levels of 4.0–10.0 ng/ml or 10.1–20.0 ng/ml (p = 0.391 and 0.386, respectively). The results of other age groups were similar to patients who underwent all four types of biopsy protocols (S2 Table).

### Impact of age on the diagnostic performance of %fPSA in predicting high-grade prostate cancer (HGPCa)

The ability of %fPSA to predict HGPCa was similar to its ability to predict any prostate cancer in each age group. %fPSA significantly outperformed PSA in men aged 70–79 years with a PSA level of 4.0–10.0 ng/ml or 10.1–20.0 ng/ml. The AUC of %fPSA also outperformed PSA in men aged 60–69 years with a PSA level of 10.1–20.0 ng/ml. The AUC of %fPSA was higher than that of PSA in other age groups, but the benefit failed to reach statistical significance.

### Impact of age on the diagnostic performance of PSA + %fPSA PCa or HGPCa

The diagnostic accuracy improvement was minor when PSA and %fPSA were both incorporated in the PCa prediction (Table 2). Neither %fPSA nor PSA + %fPSA outperformed PSA alone in men aged 50–59 years with a PSA level of 4.0–10.0 ng/ml or 10.1–20.0 ng/ml. The combination of %fPSA and PSA improved the AUC of %fPSA in other age groups, but the improvements were minor. The ability of PSA + %fPSA to predict HGPCa was similar to the ability of PSA +%fPSA to predict any prostate cancer, but no significant improvements were conferred by combining PSA with %fPSA (Table 2).

### Identification of the proper age range for the application of %fPSA

We categorized patients into six age groups with intervals of five years. The AUC of %fPSA in the PSA range 4.0–10.0 ng/ml was significantly better than PSA in patients aged 75–79 years (p = 0.024), and 70-74-year-olds were borderline significant (p = 0.085). However, %fPSA improved the AUC of PSA in all age ranges except men aged 50–59 years with a PSA range of 10.1–20.0 ng/ml. The highest AUC was 0.673 in patients aged 65–69 years.

### Age-specific %fPSA reference range for patients with PSA levels of 4.0–10.0 ng/ml indifferent age groups

Different %fPSA cutoff values were evaluated in patients with PSA levels of 4.0–10.0 ng/ml. Sensitivity, specificity, positive predicted value (PPV) and negative predicted value (NPV) were calculated at all possible cutoff values. The highest NPV were 86.6%, 79.5% and 76.1%, respectively for patients aged 60–69, 70–79 and ≥80 years, respectively. We failed to identify a %fPSA value above which the likelihood of detecting PCa is minimal (less than 10%). Nevertheless, if our aim was to detect the dominant majority of PCa (90%), while reduce unnecessary biopsies, we identified age-specific cutoff values to reduce unnecessary biopsies on the basis of maintaining the detection of 90% of all PCa patients in that age group. The cutoff values were 24%, 27% and 32% for patients aged 60–69, 70–79 and ≥80 years, respectively (Table 3). The NPV at these cutoff values were 85.4%, 78.9% and 66.7% for patients in these three age groups, respectively. For instance, 19.1% of unnecessary biopsies would be avoided when the 27% cutoff was used in patients aged 70–79 years, while maintaining the detection of 89.6% of all prostate cancers patients in this age group. This strategy would only miss 37 out of 354 cases of all PCa patients. There were 23 patients diagnosed with HGPCa in the 37 patients who were missed. In contrast, the application of the reported cutoff 25% would miss more than 10% of PCa patients aged 70–79 and 80–89 years.

**Table 3:** Application of age-specific %fPSA cutoffs to reduce unnecessary biopsies in different age decades.

Age (years)	No. Pts	%fPSA cutoff	Sensitivity,%	95% CI	Specificity, %	95% CI	PPV, %	NPV, %	Missed PCa by Gleason score		
			(No. of PCa detected/all PCa)		(No. of unnecessary biopsies avoided/all negative biopsy)				≤6	7	≥8
**Maintaining the detection of 90% PCa patients**											
60–69	1193	24%	90.0 (226/251)	85.6 - 93.5	15.4 (141/942)	13.1 - 17.9	21.7	85.4	(6/24)	(10/24)	(8/24)
70–79	1051	27%	89.6 (317/354)	85.9 - 92.5	19.1 (128/697)	16.2 - 22.2	36.2	78.9	(14/37)	(11/37)	(12/37)
80–89	190	32%	88.9 (64/72)	79.3 - 95.1	9.2 (11/119)	4.7 - 15.9	37.8	66.7	(3/5)	(0/5)	(2/5)
**Reported cutoff value of 25%**											
60–69	1193	25%	91.2 (229/251)	87.0 - 94.4	11.6 (112/942)	9.6 - 13.8	21.3	83.6	(5/20)	(9/20)	(6/20)
70–79	1051	25%	86.2 (317/354)	82.1 - 89.6	22.0 (115/697)	18.9 - 25.2	36.1	75	(16/49)	(19/49)	(14/49)
80–89	190	25%	83.3 (60/72)	72.7 - 91.1	26.1 (31/119)	18.4 - 34.9	40.3	72.1	(6/12)	(0/12)	(6/12)

No. Pts: number of patients.

The fixed cutoff of 25% for the PSA range 10.1–20.0 ng/ml yielded a detection rate of 90.2% to 93.4% in patients aged 60–69, 70–79 and ≥80 years. This cutoff would reduce unnecessary biopsies by 17.2% in patients aged 70–79 years while compromising sensitivity by only 9.8%. A cutoff of 25% would avoid 29.0% of unnecessary biopsies in patients aged 80–89 years while maintaining a PCa detection rate of 90.4%. A cutoff value for each age group was not necessary in this circumstance.

## Discussion

### Application of %fPSA in Chinese men

%fPSA is an important predictor of PCa in patients with a PSA level of 4.0–10.0 ng/ml However, there are huge unexplained differences between races. For instance, the European Association of Urology Guidelines indicated that %fPSA was not applicable for patients with a PSA level >10 ng/ml. However, we demonstrated that %fPSA was not more effective than PSA in younger patients (<60 years), and %fPSA also improved the diagnosis of PCa in PSA ranges of 10.1–20.0 ng/ml and 4.0–10.0 ng/ml in Chinese men in our previous studies. To translate the results in the ROC curve analysis to a binary and clinical applicable test, we tried to identify the specific cutoff values to reduces unnecessary biopsies using the same data set. The underlying reason for all of these findings may be the lower PCa detection rate at the same PSA level in Chinese compared with Western patients. For instance, the PCa detection rate of patients with PSA levels of 4.0–10.0 ng/ml was 25.1% in this Chinese cohort, but it was 40.3% and 43.4% in consecutive clinical patients in the Cleveland Clinic and Durham Veteran’s Affair Hospital, respectively, and 34.8% and 41.4% in the Tarn and SABOR screening programs, respectively [15, 16]. The PCa detection rate in Korean patients with PSA levels of 4.0–10.0 ng/ml was only 17.0% [11], and it was approximately 20–25% in Japan, Hong Kong, Singapore and India as reported by a cooperative study of Asian urologists [17]. The detection rate of PCa in Chinese men with a PSA level of 10.1–20.0 ng/ml was 36.5%, which is similar to the Western population with a PSA level of 4.0–10.0 ng/ml. Thus, the effectiveness of %fPSA in Chinese patients with a PSA level of 10.1–20.0 ng/ml may be similar to the Western population with a PSA level of 4.0–10.0 ng/ml.

### Racial differences in PSA between Chinese and Western populations

Chinese, Japanese, Korean and other Asian populations share a similar incidence rate and genetic and social environments. Therefore, the findings of this study may offer a hint for further studies in these countries [18]. However, whether this disparity was the result of genetic or environmental differences is not known. Recently, Chinese researchers identified a novel genetic variant in the SLC45A3 gene that was associated with the level of serum PSA in a genome-wide association study (GWAS) [19]. Their discovery was confirmed in a study of a Japanese population [20]. A GWAS in a European population showed that a total of six loci were associated with PSA level [21], but two of them were not confirmed in Asian populations. Indeed, PSA was first identified and validated in a Caucasian population, and it was confirmed in Chinese and other Asian populations [22]. We suggest that the effectiveness and proper manner of using %fPSA be further evaluated in China and other Asian countries.

### Why are age-specific %fPSA reference ranges recommended in Chinese but not Western populations?

There is great ethno-geographical variation in the prevalence of prostate cancer. The differences in PSA reference range and detection rate of the same PSA level are significantly different between different races. Our findings illustrate that age-specific cutoff values are helpful to reduce unnecessary biopsies, but the influence of age should also be considered. Multiple age-specific cutoff values, instead of a single cutoff value, should be considered for men with a PSA level of 4.0–10.0 ng/ml to maintain the same level of sensitivity. Increasing numbers of urologists realize that a lower %fPSA poses a continuous risk for PCa similarly to rising PSA. Therefore, we put particular emphasis on the impact of age on the diagnostic accuracy of %fPSA.

Previous studies in Western populations identified age-specific cutoffs for %fPSA, but these values were not strongly recommended. Catalona et al. [5] demonstrated that different cutoff values should be used in different age groups to yield the same sensitivity of 95% (detect 95% of all PCa patients). According to this method of calculation, the proposed cutoff values were 20%, 26% and 28% in patients aged 50–59, 60–69 and 70–75 years, respectively, for men with a PSA level of 4.0–10.0 ng/ml. Their study illustrated that higher cutoff values would fit older patients. However, the age-specific cutoffs were not strongly recommended because the single cut of 25% would yield sensitivities of 90%, 92% and 96% in patients aged 50–59, 60–69 and 70–75 years, respectively. This cutoff delivers higher sensitivity in younger patients, which is more desirable because these patients are more likely to benefit from aggressive and curative therapies. Nevertheless, the diagnostic performance of %fPSA in Chinese patients with a PSA level of 4.0–10.0 ng/ml was not as good as that in Western populations. However, this cutoff was not applicable in a Chinese population because 13.8% (37/354) and 16.7% (12/72) of PCa patients aged 70–79 and 80–89 years, respectively, were missed. Notably, a proper cutoff value of %fPSA was also proposed in a Turkish population to avoid unnecessary biopsies [23]. However, the age-specific cutoff values were 10%, 15% and 15% in patients aged 50–59, 60–69 and >70 years, respectively, which are much lower than our study and reports in Western populations. The author attributed these differences to the different assays applied [23].

### Why are age-specific %fPSA cutoff values recommended for patients with a PSA level of 4.0–10.0 ng/ml but not 10.1–20.0 ng/ml?

We did not emphasize the application of age-specific cutoffs for patients with a PSA level of 10.1–20.0 ng/ml, although these cutoff values were also identified. First, the AUC of %fPSA was higher in patients with a PSA level of 10.1–20.0 ng/ml than patients with a PSA level of 4.0–10.0 ng/ml. The single cutoff of 25% would maintain a sensitivity of over 90% in all age decades. Second, the detection rate of Chinese patients with a PSA level of 10.1–20.0 ng/ml (36.4%) was similar to consecutive Western patients with PSA levels of 4.0–10.0 ng/ml from outpatient departments [15, 16]. The single cutoff performed well and maintained high sensitivity in reducing unnecessary biopsies in both of groups of patients.

### Potential bias of this study

Several circumstances must be considered when drawing conclusions from this data set. First, there is an inherent limitation of multicenter studies because the biopsies were performed by different physicians and examined by different pathologists. Second, 22 institutes and three different assays were involved in PSA testing. However, the variability of the total and fPSA results between the commercial assays was diminished by calibration. Therefore, these findings should be validated in prospective multicenter studies. Despite this caveat, our data depict the effectiveness of %fPSA in a practical setting for Chinese men. These findings should be validated in prospective multi-center studies. Finally, the age-specific cutoff values were proposed on the basis of maintaining the detection of maximal prostate cancer patients (over 90% of all PCa patients detected), however, we failed to propose these cutoff values to ensure that all the patients were excluded from biopsy would have a minimal chance of PCa (more than 90% of the patients who were determined to not to undergo biopsy were negative). This drawback should be fully acknowledged in applying this strategy.

## Conclusions

Our results indicate that age has a strong impact on %fPSA and its diagnostic performance in predicting prostate biopsy outcomes in Chinese men with a PSA level of 4.0–10.0 ng/ml or 10.1–20.0 ng/ml. We do not recommend the use of a fixed cutoff for all clinical patients, but we emphasize the importance of taking age into consideration when using %fPSA to reduce unnecessary biopsies.

## Supporting Information

S1 Table Diagnostic accuracy of total PSA and %fPSA in predicting prostate cancer stratified by prostate volume, biopsy schemes and biopsy path. (DOC)

S2 Table Diagnostic accuracy of total PSA and %fPSA in predicting prostate cancer in patients underwent extended biopsy by age groups. (DOCX)
