# Supplementary material for: Age-Specific Cutoff Value for the Application of Percent Free Prostate-Specific Antigen (PSA) in Chinese Men with Serum PSA Levels of 4.0–10.0 ng/ml
Source: PLoS One. 2015 Jun 19;10(6):e0130308. doi: 10.1371/journal.pone.0130308 (PMC4474838; doi:10.1371/journal.pone.0130308)
Supplement: S1 Table — (DOC) [file pone.0130308.s001.doc]

**S1 Table Diagnostic accuracy of total PSA and %fPSA in predicting prostate cancer stratified by prostate volume, biopsy schemes and biopsy path.**

|  | No. Pts | AUC | | P* | No. Pts | AUC | | P* |
| --- | --- | --- | --- | --- | --- | --- | --- | --- |
|  |  | PSA | %fPSA |  |  | PSA | %fPSA |  |
|  | PSA 4.0-10.0ng/ml | | | | PSA 10.1-20.0ng/ml | | | |
| PV (ml): |  |  |  |  |  |  |  |  |
| < 30 | 603 | 0.595 (0.548 - 0.642) | 0.511 (0.464 - 0.559) | 0.017 | 440 | 0.569 (0.516 - 0.623) | 0.570 (0.516 - 0.624) | 0.991 |
| 30–39.9 | 563 | 0.534 (0.483 - 0.586) | 0.522 (0.471 - 0.573) | 0.719 | 418 | 0.546 (0.490 - 0.602) | 0.497 (0.441 - 0.546) | 0.210 |
| 40–60 | 756 | 0.508 (0.458 - 0.558) | 0.503 (0.454 - 0.551) | 0.890 | 586 | 0.550 (0.501 - 0.598) | 0.574 (0.526 - 0.621) | 0.480 |
| >60 | 640 | 0.489 (0.431 - 0.548) | 0.514 (0.452 - 0.576) | 0.568 | 763 | 0.559 (0.506 - 0.613) | 0.559 (0.507 - 0.611) | 0.998 |
| Biopsy schemes: |  |  |  |  |  |  |  |  |
| Sextant | 263 | 0.549 (0.468 - 0.629) | 0.551 (0.473 - 0.629) | 0.969 | 215 | 0.592 (0.515 - 0.668) | 0.664 (0.591 - 0.736) | 0.202 |
| 8-core | 589 | 0.532 (0.478 - 0.586) | 0.577 (0.523 - 0.631) | 0.221 | 456 | 0.545 (0.489 - 0.600) | 0.625 (0.572 - 0.677) | 0.038 |
| 10-core | 824 | 0.540 (0.490 - 0.590) | 0.565 (0.514 - 0.616) | 0.485 | 834 | 0.530 (0.487 - 0.573) | 0.617 (0.577 - 0.657) | 0.002 |
| 12-core | 1461 | 0.535 (0.502 - 0.567) | 0.562 (0.530 - 0.594) | 0.083 | 1203 | 0.549 (0.515 - 0.583) | 0.594 (0.560 - 0.627) | 0.063 |
| Biopsy Path: |  |  |  |  |  |  |  |  |
| TURS-guided | 2613 | 0.534 (0.509 - 0.559) | 0.558 (0.533 - 0.583) | 0.159 | 2239 | 0.567 (0.539 - 0.589) | 0.621 (0.597 - 0.646) | 0.001 |
| Transperineal | 548 | 0.534 (0.476 - 0.592) | 0.535 (0.477 - 0.592) | 0.983 | 515 | 0.510 (0.458 - 0.561) | 0.581 (0.530 - 0.631) | 0.068 |

No. Pts: number of patients.

* z test.
