# Supplementary material for: Age-Specific Cutoff Value for the Application of Percent Free Prostate-Specific Antigen (PSA) in Chinese Men with Serum PSA Levels of 4.0–10.0 ng/ml
Source: PLoS One. 2015 Jun 19;10(6):e0130308. doi: 10.1371/journal.pone.0130308 (PMC4474838; doi:10.1371/journal.pone.0130308)
Supplement: S2 Table — (DOCX) [file pone.0130308.s002.docx]

**S2 Table Diagnostic accuracy of total PSA and %fPSA in predicting prostate cancer in patients underwent extended biopsy by age groups.**

| Age, | No. Pts | AUC | | P* | No. Pts | AUC | | P* |
| --- | --- | --- | --- | --- | --- | --- | --- | --- |
| (years) |  | PSA | %fPSA |  |  | PSA | %fPSA |  |
|  | PSA 4.0-10.0ng/ml | | | | PSA 10.1-20.0ng/ml | | | |
| 50-59 | 331 | 0.549(0.463 - 0.634) | 0.503(0.418 - 0.587) | 0.391 | 205 | 0.525(0.412-0.637) | 0.589(0.485-0.692) | 0.386 |
| 60-69 | 869 | 0.548(0.501 - 0.596) | 0.585 (0.538 - 0.632) | 0.259 | 725 | 0.527(0.481 - 0.572) | 0.619(0.573 - 0.665) | 0.002 |
| 70-79 | 768 | 0.518(0.475-0.561) | 0.629(0.587-0.670) | <0.001 | 798 | 0.525(0.484 - 0.567) | 0.631(0.591 - 0.671) | <0.001 |
| 80-89 | 128 | 0.562 (0.461-0.663) | 0.588(0.486-0.689) | 0.708 | 195 | 0.577(0.497-0.658) | 0.687(0.611 - 0.763) | 0.047 |
